# Ionic Liquids — Promising but Challenging Solvents for Homogeneous Derivatization of Cellulose

**DOI:** 10.3390/molecules17067458

**Published:** 2012-06-15

**Authors:** Martin Gericke, Pedro Fardim, Thomas Heinze

**Affiliations:** 1 Laboratory of Fibre and Cellulose Technology, Åbo Akademi University, Porthansgatan 3 FI-20500 Turku, Finland; Email: mgericke@abo.fi (M.G.); pfardim@abo.fi (P.F.); 2 Institute of Organic Chemistry and Macromolecular Chemistry, Friedrich Schiller University of Jena, Centre of Excellence for Polysaccharide Research, Humboldtstraße 10, D-07743 Jena, Germany

**Keywords:** cellulose, ionic liquids, homogeneous synthesis, cellulose derivatives, reaction media

## Abstract

In the past decade, ionic liquids (ILs) have received enormous interest as solvents for cellulose. They have been studied intensively for fractionation and biorefining of lignocellulosic biomass, for dissolution of the polysaccharide, for preparation of cellulosic fibers, and in particular as reaction media for the homogeneous preparation of highly engineered polysaccharide derivatives. ILs show great potential for application on a commercial scale regarding recyclability, high dissolution power, and their broad structural diversity. However, a critical analysis reveals that these promising features are combined with serious drawbacks that need to be addressed in order to utilize ILs for the efficient synthesis of cellulose derivatives. This review presents a comprehensive overview about chemical modification of cellulose in ILs. Difficulties encountered thereby are discussed critically and current as well as future developments in this field of polysaccharide research are outlined.

## 1. Introduction

**Figure 1:**
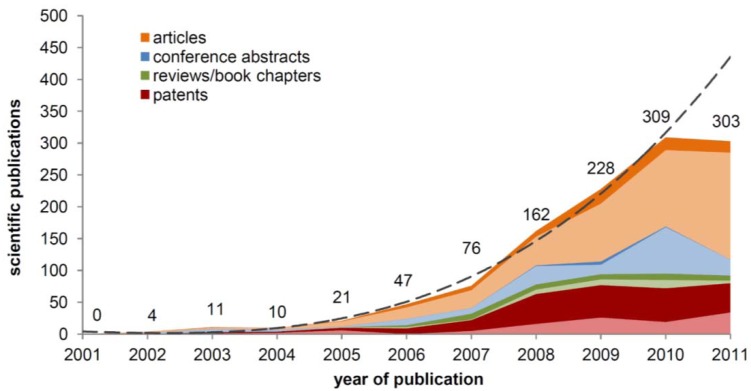
Publications in the years 2001 to 2011, found in the SciFinder^®^ database for the term “cellulose ionic liquids”, separated by document type (bright/dark areas correspond to articles published in English/other languages). Dotted lines represent a prognoses based on the number of total publications (excluding data for 2011).

In 2002 it has been reported that certain ionic liquids (ILs), molten organic salts with a melting point below 100 °C, can dissolve high amounts of cellulose (≈10–20%) within short times (1–12 h) and without any pretreatment [1]. Since that time, an increasing number of scientific papers, patents, review articles, and conference abstracts about the use of ILs as cellulose solvents has been published and ILs have become one of the “hot-topics” in polysaccharide research (Figure 1) [2,3,4]. 

ILs have been exploited for shaping of cellulose into fibers, films, sponges, beads, and other cellulosic objects [5,6,7,8,9]. In addition, they are studied intensively for “biorefinery applications”, which includes fractionation of lignocellulosic biomass, IL-pretreatment of cellulose for improving enzymatic hydrolysis, and conversion of cellulose dissolved in ILs into mono/disaccharides, platform chemicals, and biofuels. These complex aspects have already been reviewed separately and are consequently not part of the present work [10,11,12].

One of the most promising applications of cellulose dissolving ILs and topic of the present work is their use as reaction media for the homogeneous preparation of highly engineered polysaccharide derivatives. A vast number of organic reactions have been performed in ILs with high efficiency and, although very common in low-molecular chemistry, most of these advanced syntheses are still waiting to be exploited in cellulose chemistry [13]. Task specific ILs can be prepared and tailored for particular fields of application [14,15]. Moreover, ILs might overcome the limitation of other cellulose solvents, used so far for the homogeneous derivatization, regarding recyclability and cost efficiency. They are intensively studied for the production of commercial bulk derivatives, such as cellulose esters, e.g., acetates, propionates, butyrates, and mixed esters [16,17,18,19,20,21,22]. These derivatives are still prepared under heterogeneous starting conditions despite the fact that solvents for the homogeneous esterification of cellulose in lab-scales are well known and proved to be advantageous in terms of efficient control of amount and distribution of substituents along the polysaccharide backbone [23,24].

Although ILs proved to be very promising polysaccharide solvents, they are likewise very challenging to work with. They show certain drawbacks that need to be addressed before ILs can be applied for the dissolution and processing of cellulose on a commercial scale. After almost 10 years of research in this field, it is justified to make a critical evaluation in order to promote future developments. The fact that in 2011 the number of scientific publications in that field fell behind expectation and stagnated on the level of 2010 might be an indication that research in this area is at a tipping point. Aim of the present article is to provide comprehensive information on recent, current, and future developments regarding the use of ILs as solvents for the homogeneous derivatization of cellulose. Difficulties and drawbacks encountered thereby are summarized and likewise opportunities are outlined regarding the use of ILs for the synthesis of highly engineered polysaccharide derivatives. 

## 2. History

### 2.1. Dissolution of Cellulose in Molten Organic Salts

The first descriptions on the use of organic salts with a low melting point as solvents for cellulose date back to the early 1930s [25,26]. *N*-Alkylpyridinium chlorides have been used therein for the dissolution and benzylation of cellulose. These compounds cannot be defined as true ILs because they melt around 20–30 K above the 100 °C limit. Small amounts of pyridine were required in order to decrease the melting point and achieve dissolution of cellulose. Mixtures of pyridinium salts with co-solvents such as dimethylsulfoxide (DMSO) or *N*,*N*-dimethylformamide (DMF) have been applied for dissolution, derivatization, and shaping of cellulose to spherical beads [27,28]. None of these discoveries attracted significant attention during that time. Nevertheless, they deserve to be mentioned for the sake of completeness and also because these publications demonstrated the beneficial use of co-solvents (see Section 4.2.).

**Figure 2:**
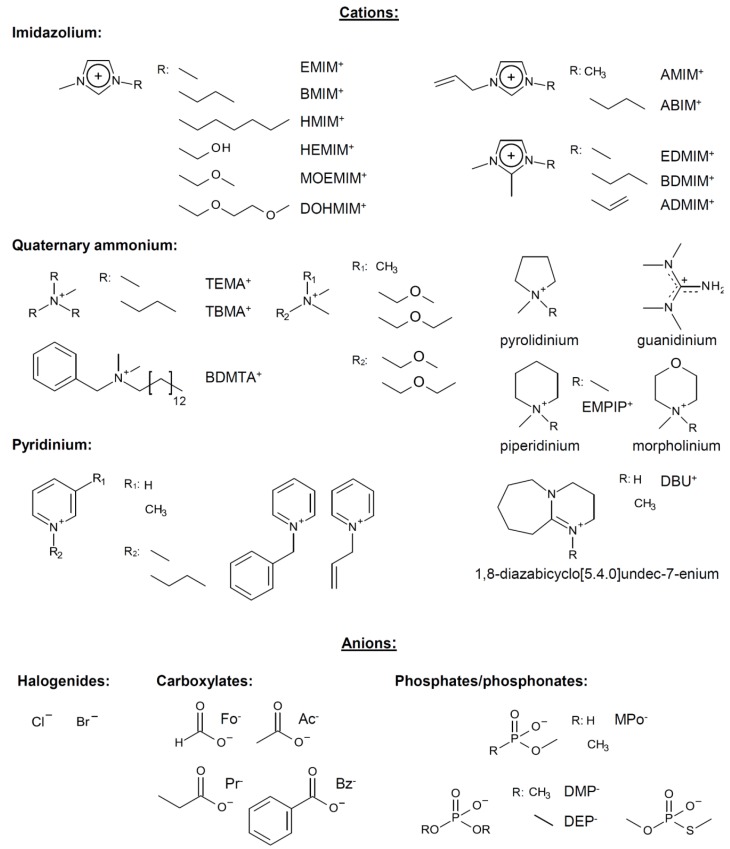
Molecular structures and abbreviations of anions and cations of typical ionic liquids and low-melting organic salts, reported for dissolution of cellulose.

In the past decades, ILs have been studied intensively as novel solvents for various applications in electrochemistry, analytical chemistry, and organic synthesis [13,29,30,31]. Within the framework of this increasing interest, dissolution of cellulose in 1-butyl-3-methylimidazolium chloride (BMIMCl) and other “real” ILs, *i.e.*, undiluted molten organic salts with melting points below 100 °C, have been described in 2002 [1]. In the following years, more cellulose-dissolving ILs have been reported [2,3,4,32]. The molecular structures of the most frequently used ones are displayed in Figure 2. The majority are based on dialkylimidazolium cations but pyridinium- and quaternary ammonium salts have also been reported. The most frequently reported anions are chloride and acetate. In addition, ILs bearing other anions such as carboxylates, alkyl phosphate, and alkyl phosphonates have been utilized as cellulose solvents [32]. It has to be pointed out that the ability to dissolve cellulose is not an inherent property of this broad class of compounds. Among the vast number of ILs reported so far, only a minority dissolve cellulose. In particular the anions are restricted to a small number of suitable ones. For the ease of reading, however, the term “IL”, used in the following passages, refers to those that are able to dissolve cellulose, if not specified otherwise.

### 2.2. Ionic Liquids as Reaction Media for Cellulose Derivatization

Cellulose contains crystalline as well as amorphous regions that show different reactivity towards heterogeneous conversion [33]. Pretreatment procedures, e.g., stepwise solvent exchange or alkali treatment, are usually applied in order to avoid the formation of inhomogeneous product mixtures, consisting of highly substituted and almost unmodified parts, and to obtain products with a high degree of substitution (DS). Dissolution of cellulose usually increases its reactivity. Homogeneous derivatization of cellulose enables easy control of the total amount of functional groups and of their distribution along the polymer chain [23,24]. Various cellulose solvents, such as *N*,*N*-dimethylacetamide (DMA)/LiCl, DMSO/tetra-*N*-butylammonium fluoride (TBAF), and molten inorganic salt hydrates have been applied successfully for homogeneous derivatization of cellulose [32,33,34,35]. Although they have been studied intensively for a long time, utilization of these solvents is restricted to lab-scale syntheses, mostly due to their high costs and poor recyclability. ILs might overcome these limitations and eventually facilitate the homogeneous preparation of novel cellulose derivatives. An overview on cellulose derivatives that have been prepared in different ILs, is presented in Table 1, including the range of DS values obtained. If not noted otherwise, these reactions proceeded completely homogeneously. In the following passage, different types of derivatization reactions in ILs are described in detail. Afterwards, difficulties that might hamper future use of ILs as solvents in cellulose chemistry are discussed.

**Table 1:** Overview over cellulose derivatives, in ionic liquids (ILs).

Entry	Substituent (Cell-OR)	DS ^a^	ILs ^b^	Comments ^c^	Ref.
	carbamates				
1	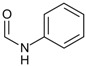 phenyl carbamate	0.5–3.0	BMIMCl		[36]
		0.3–3.0	BMIMCl	- derivatization of bacterial cellulose	[37]
2	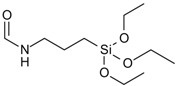 3-(triethoxysilyl)propyl carbamete	0.43, 2.95	BMIMCl	- hydrolysis of ethoxysilyl groups directly after synthesis	[38]
	carboxylic acid esters				
3	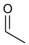 acetate	0.9–2.8	AMIMCl		[39,40]
		1.9–3.0	BMIMCl, EMIMCl, BDMIMCl, ADMIMBr		[36,41]
		0.7–3.0	BMIMCl	- derivatization of bacterial cellulose	[37]
4	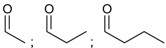 propionate/butyrate/… and mixed esters	≈0.5–2.9	ABIMCl, BMIMAc, DOHMIMAc, HMIMAc, MOEMIMAc	- cellulose esters and mixed esters prepared- microwave irradiation (partly)- also pentanoates and hexanoates prepared	[42,43,44]
		1.4–2.7(DS_overall_)	AMIMCl	- acetate-butyrates and acetate-propionates prepared- derivatization of cellulose from sugarcane bagasse	[45]
		0.5–2.9(DS_overall_)	BMIMAc, BMIMCl, BMIMDMP, BMIMPr, EMIMAc, TBMADMP	- cellulose esters and mixed esters prepared- co-solvents applied (partly)- also mixed esters with benzoate prepared	[16,17,18,19,20]
		0.2–2.5	BMIMCl, EMIMAc	- heterogeneous conversion with gaseous ketenes- acetates, propionates, and pentanoates prepared	[21]
5	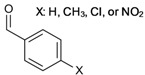 benzoates	1.0–3.0	AMIMCl		[46]
6	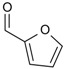 fuorate	0.5–3.0	BMIMCl	- activation of carboxylic acid with N,N’-carbonyldiimidazole (partly)	[47]
7	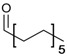 laurate	0.3–1.5	BMIMCl	- phase separation with increasing DS	[36]
8	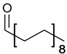 stearate	2.2–2.6	BMIMCl		[48]
9	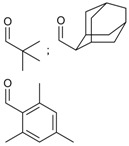 pivalate, adamantate, trimethylbenzoate	0.9–1.4	AMIMCl	- comparison with DMA/LiCl and DMSO/TBAF	[49]
10	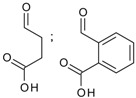 succinate, phthalate	0.2–2.5	AMIMCl, BMIMCl	- derivatization of cellulose from sugarcane bagasse- catalysts applied - co-solvents utilized (partly)	[50,51,52,53]
11	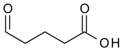 glutarate	0.3–1.2	BMIMCl	- ultrasound irradiation	[54]
12	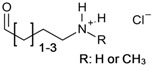 carboxylates containing amino groups	0.1-1.2	BMIMCl	- tosyl chloride used to form reactive intermediates- co-solvents utilized	[55,56]
13	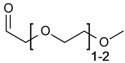 oxy-carboxylic acid esters	0.1–3.0	AMIMCl, BMIMCl, EMIMCl	- activation of oxy-carbonic acid with N,N’-carbonyldiimidazole- derivatization of bacterial cellulose (partly)	[57]
14	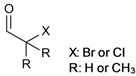 2-halo carboxylate macro-initiators	0.6–1.0	AMIMCl	- co-solvents utilized- bromo compounds utilized	[58,59,60]
		0.3–1.9	BMIMCl	- chloro compounds utilized	[61]
15	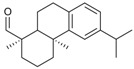 dehydroabiatate	1.4–1.9	BMIMBr	- catalyst applied	[62]
16	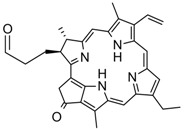 *pyro*-pheophorbide	0.07	AMIMCl	- activation of acid with N,N'-carbonyldiimidazole	[63]
	ethers				
17	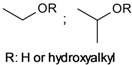 hydroxethyl/hydroxypropyl	0.1–2.2	BMIMCl, BDMIMCl, BDTAC, EMIMAc	- co-solvents utilized	[64,65]
18	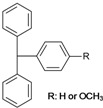 trityl/methoxytrityl	0.8, 1.8	AMIMCl	- pyridine utilized as base and co-solvent	[66]
		0.8–1.4	BMIMCl	- pyridine utilized as base and co-solvent	[67]
19	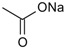 carboxymethyl	0.49	BMIMCl	- co-solvents utilized- heterogeneous (solid NaOH used as base)- gel-like system formed	[36]
		n.a.	BMIMCl	- heterogeneous (solid NaOH used as base)	[68]
20	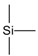 trimethylsilyl	0.4–2.9	BMIMCl, EMIMAc	- co-solvents utilized	[69]
		0.2–3.0	BMIMCl, BMIMAc, BMIMBz, BMIMPr, EMIMAc, EMIMDEP	- heterogeneous derivatization (polar and non-polar liquid phase)	[70]
	sulfuric/sulfonic acid esters				
21	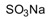 sulfate	0.1–1.5	AMIMCl, BMIMCl, EMIMCl	- co-solvents utilized	[71,72]
		1.3–1.7	BMIMCl	- co-solvents utilized	[73]
22	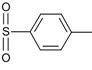 tosylate	0.1–1.1	AMIMCl, BMIMCl	- co-solvents utilized, reaction at 25 °C	[74]
		0.84	AMIMCl	- reaction at 10 °C	[63]
	deoxy cellulose derivatives				
23	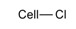 chloro-deoxy	0.8–1.l	BMIMCl	- co-solvent utilized- strong polymer degradation	[75]
	grafts				
24	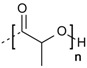 *graft*-poly(L-lactide)	0.7–2.7(1.4–4.5) ^d^	AMIMCl	- grafting by ring-opening of L-lactide- 4-dimethylamino pyridine applied as catalyst	[76]
25	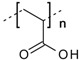 *graft*-poly(acrylic acid)	n.a.	BMIMCl	- initiation by persulfate - microwave irradiation	[77]
26	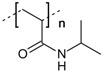 *graft*-poly(*N*-isopropylacrylamide)	n.a.	BMIMCl	- initiation by γ-ray irradiation	[78]
27	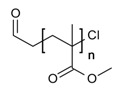 graft-poly(methyl methacrylate)	0.3–1.9	BMIMCl	- derived from 14 by atom transfer radical polymerization	[61]

^a^ Range of degrees of substitution (DS), n.a.: no information available; ^b^ Cations: ABIM^+^: 1-allyl-3-butylimidazolium, ADMIM^+^: 1-allyl-2,3-dimethylimidazolium, AMIM^+^: 1-allyl-3-methylimidazolium, BDMIM^+^: 1-butyl-2,3-dimethylimidazolium, BMIM^+^: 1-butyl-3-methylimidazolium, BDMTA^+^: benzyldimethyltetradecylammonium, DOHMIM^+^: 1-(3,6-dioxa-(1-heptyl))-3-methylimidazolium, EDMIM^+^: 1-ethyl-2,3-dimethylimidazolium, EMIM^+^: 1-ethyl-3-methylimidazolium, EMPIP^+^: 1-ethyl-1-methylpiperidinium, HEMIM^+^: 1-hydroxyethyl-3-methylimidazolium, HMIM^+^: 1-hexyl-3-methylimidazolium, MOEMIM^+^: 1-(2-methoxyethyl)-3-methylimidazolium, TBMA^+^: tributylmethylammonium, TEMA^+^: triethtylmethylammonium, anions: Ac^−^: acetate, Br^−^: bromide, Bz^−^: benzoate, Cl^−^: chloride, DEP^-^:diethylposphate, DEP^−^: dimethylposphate, Fo^−^: formate, MPo^−^: methylphosphonate, Pr^−^: propionate; ^c^ Completely homogeneous conversion, if not noted otherwise; ^d^ Values in braces represent degree of polymerization of the grafted chain.

#### 2.2.1. Esterification

Conversion of cellulose, dissolved in different ILs, with carboxylic acid chlorides or anhydrides proved to be very efficient for the preparation of various cellulose esters (Table 1, entries **3**–**16**). The esterifications have been carried out at elevated temperature (80–100 °C) and were found to proceed completely homogeneously, except for the syntheses of hydrophobic fatty acid derivatives with high DS. Cellulose acetates with high DS values up to 3.0, *i.e.*, completely acetylated derivatives, could be prepared within a short reaction time (0.5–8 h), with comparably small amounts of derivatization reagent (3–5 equivalents), and without using a base (entry **3**) [36,40,41]. Even bacterial cellulose, which is difficult to dissolve in other cellulose solvents because of its very high degree of polymerization (DP) up to 10,000, the high crystallinity, and comparably fine fibrils, could easily be esterificated using BMIMCl as reaction medium [37]. Acetylation of cellulose in eutectic mixtures of choline chloride and ZnCl_2_, as cheap alternative to imidazolium-based ILs, has been reported but the reactivity of the acetylating reagent was lower compared to ILs [79]. A DS of about 1 was achieved in this system using 20 equivalents of acetic anhydride while acylation of cellulose in AMIMCl and BMIMCl results in almost complete esterification (DS = 2.5–3.0) with only 3–5 equivalents.

Mixed esters have been prepared in ILs by simultaneous conversion of cellulose with anhydrides of acetic- and a second carboxylic acid (entry **4**) [42,43,45]. Cellulose acetates, as well as mixed acetate-propionates and acetate-butyrates are of great interest for the production of fibers, films, coatings, and additives used in many commercial applications [80,81]. Recently, various patents have been published by Eastman Chemical Company that describe the homogeneous preparation of cellulose esters and mixed esters, the synthesis of the imidazolium- and ammonium-based ILs used, and the recycling of the ILs by evaporation of precipitation agent and residues of the volatile acylation reagents [16,17,18,19,20]. In addition, heterogeneous esterification of cellulose, dissolved in ILs, using gaseous ketenes has been patented by BASF [21].

Cellulose, dissolved in 1-allyl-3-methylimidazolium chloride (AMIMCl), could be converted with bulky acid chlorides (entry **9**) showing some regioselectivity at DS of about 0.9–1.4 [49]. Results obtained for acylation in the IL were similar to conversions in DMA/LiCl and DMSO/TBAF although a decrease in reactivity was observed in the latter case. Long chain fatty acid esters of cellulose (entry **7**–**8**) with high DS up to 2.6 have been prepared in ILs but not under completely homogeneous conditions. It has been reported that upon advancing substitution, cellulose laurate precipitates from the IL reaction medium because it becomes increasingly hydrophobic [36]. Although not explicitly stated in the corresponding reference, the same can be expected for the even more hydrophobic cellulose stearates [48].

Cellulose benzoates with different moieties at the aromatic ring (entry **5**) have been prepared homogeneously in AMIMCl by conversion of cellulose with the corresponding benzoyl chlorides [46]. These materials with relatively high DS values between 1.0 and 3.0 might find use as chiral stationary phase in chromatography. IL/DMF mixtures have been exploited as reaction media for the homogeneous esterification of cellulose with haloacyl halogenides [58,59,60,61]. The 2-bromo- and 2-chloro carboxylic acid esters obtained (entry **14**) have been used as macro-initiators for grafting poly(methacrylates) and poly(styrene) onto the cellulose backbone via atom transfer radical polymerization (ATRP).

AMIMCl and BMIMCl have been applied as reaction media for the homogeneous preparation of dicarboxylic acid esters like cellulose phthalates and cellulose succinates (entry **10**) [50,51,52,53]. *N*-Bromosuccinimide, 4-dimethylamino pyridine (DMAP), or iodine were utilized as catalysts and in most cases DMSO was added as co-solvent in order to obtain homogenous reaction mixtures. Cellulose dehydroabietates (entry **15**) have been prepared by DMAP catalyzed esterification in 1-butyl-3-methylimidazolium bromide (BMIMBr) [62]. The acid chloride used has been prepared in a previous conversion of the corresponding acid with thionyl chloride. Carboxylic acids are usually more readily available but show significantly lower reactivity compared to their anhydride or chloride derivatives. A very elegant method to activate carboxylic acids for the esterification of cellulose is the conversion into imidazolides [24,82]. These reactive intermediates, which further react with the polysaccharide, are prepared *in situ* in ILs by conversion of the corresponding carboxylic acid with *N*,*N'*-carbonyldiimidazole (CDI), only CO_2_ and imidazole are formed as side products. Activation of carboxylic acids with CDI in ILs is particularly useful for obtaining cellulose esters with high DS up to 3.0 under mild reaction conditions. Via this procedure, cellulose fuorates (entry **6**) and water soluble oxy-carboxylic acid esters of cellulose (entry **13**) with a broad range of DS values from 0.1 to 3.0 could be prepared in ILs [47,57]. The preparation of a cellulose *pyro*-pheophorbide derivative with DS = 0.07 (entry **16**) by activation of the corresponding acid with CDI has also been reported [63].

The combination of ILs, as novel solvents, and microwaves, as an efficient energy source, has been studied intensively for numerous organic syntheses, including cross-coupling reactions and olefin metathesis. Compared to conventional heating, microwave irradiation was found to result in increased yields and significant reduction of reaction times, from several hours to several minutes [83]. Cellulose dissolution in ILs with the aid of microwave heating has been reported frequently as well [1,84,85]. Microwave assisted acetylation of cellulose in 1-allyl-3-butylimidazolium chloride (ABIMCl) has been reported to yield a higher DS of 2.8 compared to the derivatization under conventional heating (DS = 2.2; reaction conditions in both cases: 8 h, 80 °C, 4.5 equivalents acetic anhydride) [43,44]. Grafting of acrylic acid onto cellulose, dissolved in BMIMCl, has been performed under microwave irradiation as well [77]. Due to their ionic character, ILs rapidly heat within a microwave field [86]. As a result of the high viscosity of ILs, efficient stirring during microwave irradiation is necessary to distribute the heat evenly and to avoid local spots of extremely high temperature. This aspect becomes even more vital for highly viscose cellulose/IL solutions. In order to avoid degradation of cellulose and carbonization, which is observed by a color change of the cellulose solution from pale white/yellow to deep black, the parameters of microwave heating, e.g., power input, maximum temperature, pulse length, and pulse interval, need to be controlled precisely. In addition to the use of microwaves, the preparation of cellulose glutarate in BMIMCl under ultrasound irradiation has been described (entry **11**) [54].

In contrast to acylation of cellulose in ILs, synthesis of sulfuric and sulfonic acid esters of cellulose has been demonstrated to be more challenging because the reactions have to be carried out at room temperature, which caused insufficient mixing during the reaction (see Section 3.2.). Nevertheless, by using DMF as co-solvent to diminish the high viscosity of the imidazolium chloride-based ILs used, cellulose sulfates (entry **21**) have been prepared [71,72,73]. The derivatives showed high anticoagulant activity or could be used for the preparation of polyelectrolyte complexes, depending on the DS that ranged from 0.1 to 1.7 [73,87]. Homogeneous preparation of tosyl celluloses (entry **22**), which are important intermediates for the synthesis of various cellulose derivatives via nucleophilic displacement (S_N_) reaction, has been performed in mixtures of BMIMCl and AMIMCl with 1,3-dimethyl-2-imidazolidinone (DMI) or pyridine as co-solvents [63,74]. A procedure for the preparation of chloro-deoxy cellulose using IL/dioxane mixtures as reaction medium has been described [75]. The derivative obtained can be applied for S_N_ reaction as well. However, the strong degradation of the polymer backbone during that reaction enables only the preparation of compounds with a low DP around 25.

#### 2.2.2. Etherification

Compared to the vast number of publications that describe esterification of cellulose in ILs, reports on the preparation of cellulose ethers in these novel reaction media are scarce. Nevertheless, the first publication describing the use of ILs for the chemical derivatization of cellulose is a patent related to carboxymethylation (Table 1, entry **19**) [68]. Solid NaOH has been utilized as a base, which implies that the derivatization proceeded heterogeneously. Later it has been demonstrated that even in the presence of DMSO as a co-solvent, a gel-like mixture is formed upon addition of NaOH and the carboxymethylation reagent [41]. Therefore, only small DS values < 0.5 could be obtained.

In addition to carboxymethyl cellulose, hydroxyethyl- and hydroxypropyl celluloses (entry **17**) are among the most important ethers of cellulose that are employed in different commercial applications, e.g., as additive in paint, cement, and household products [88]. Hydroxyalkylation of cellulose with ethylene- and propylene oxide in the absence of bases has been studied in different ILs [64,65]. 1-Ethyl-3-methylimidazolium acetate (EMIMAc) has been described as the most suitable reaction medium because the acetate anion catalyzes ring opening of the oxiranes and the reaction with the polysaccharide. By adding magnesium- or potassium acetate, reasonable derivatization could be obtained also in BMIMCl as well as quaternary ammonium chlorides and formates. An increase in the degree of molecular substitution has also been observed upon the addition of DMF and DMSO, most likely because they decreased viscosity and thus favored dissolution of the gaseous oxiranes. Co-solvents are also required for the homogeneous preparation of trimethylsilyl cellulose (entry **20**). The rather hydrophilic ILs used are immiscible with the hydrophobic silylation reagents and silylated cellulose derivatives with DS > 2 [69]. Homogeneous derivatization, however, was possible in mixtures of EMIMAc and CHCl_3_; products with a DS up to 2.9 could be obtained. In addition, a procedure for heterogeneous trimethylsilylation of cellulose in different ILs has been proposed [70]. Thereby, the silylation reagent is dissolved in toluene, which is immiscible with the cellulose/IL solution and forms a separated phase in the reaction mixture. After completing the derivatization reaction, the highly substituted cellulose silylether could be isolated from the non-polar toluene phase. It is reasonable to assume that homogenous and heterogeneous trimethylsilylation in ILs result in different distribution of silyl moieties along the cellulose chain and/or within the anhydroglucose unit (AGU).

Triphenylmethyl (trityl) ethers of cellulose (entry **18**) with DS values up to 1.4 have been prepared homogeneously in BMIMCl in the presence of pyridine. [67] The bulky substituents are predominantly introduced at the primary hydroxyl group, which is more accessible compared to the sterically hindered secondary ones [89]. Thus, trityl moieties have been exploited as protection group for the C-6-position and for the regioselective synthesis of 2,3-*O*-cellulose derivatives [90,91]. By using the more reactive *p*-methoxytrityl chloride, derivatives with a higher DS of about 1.8 could be obtained in AMIMCl [66]. In order to reduce the viscosity of the reaction mixture, an excess of the base pyridine has been employed thereby.

#### 2.2.3. Other Reactions

Cellulose carbanilates (Table 1, entry **1**) with DS values up to 3.0 could be prepared in BMIMCl by conversion of celluloses of different origin, including bacterial cellulose, with phenyl isocyanate [36,37]. Carbanilation yields organosoluble derivatives and is known to proceed only with minor degradation of the polymer chain. It is consequently an important tool for determination of the molecular weight distribution of cellulose by size exclusion chromatography [92]. Moreover, no byproducts are formed during the conversion of cellulose with isocyanates. A silica hybrid material has been prepared in BMIMCl by reacting cellulose with ethoxysilyl substituted isocyanate and subsequent hydrolysis of the carbamates obtained (entry **2**) [38].

ILs have been exploited for the homogeneous preparation of different cellulose-*graft*-copolymers. Poly(L-lactide) was grafted onto cellulose (entry **24**), dissolved in AMIMCl, by DMAP catalyzed ring opening polymerization of L-lactide [76]. Cellulose-*graft*-poly(acrylamide) and -poly(accrylate) have been prepared by radical polymerization, induced by persulfate initiation or γ-ray irradiation (entry **25**–**26**) [77,78]. In the later case, the reaction was conducted under microwave heating. Cellulose-based macro-initiators (entry **14**), synthesized by homogeneous acylation in ILs, have been applied for subsequent preparation of methacrylate- and styrene *graft*-copolymers via ATRP in DMF or 1,4-dioxane [58,59,60]. No ILs or other cellulose solvents are required for the grafting reaction since the macro-initiators readily dissolve in molecular organic solvents. ILs have been used as reaction medium for ATRP as well but in that particular case, no advantages in terms of conversion rate could be observed compared to reactions in DMF [61]. ILs might be useful for grafting via ATRP, when preparation of the cellulose macro-initiator and subsequent polymerization can be combined in a one-pot synthesis without previous isolation and purification of the intermediate compound. However, the copper (I) salts that are required as catalyst might cause difficulties regarding reutilization of the ILs (see Section 3.6.).

## 3. Difficulties and Drawbacks

ILs proved to be efficient solvents for the homogeneous derivatization of a vast number of different cellulose derivatives. However, their benefits are combined with some significant drawbacks that may hamper the use of ILs as solvents for cellulose, in particular in commercialized processes. Occasionally, the disadvantages of ILs are underestimated in scientific literature but these aspects need to be addressed not only for scientific but also economic reasons. In the following passages, some important issues that have been encountered during the use of ILs as solvent and reaction medium for cellulose are discussed. Depending on the particular application or derivatization reaction, the following aspects may have different impact regarding the suitability of a particular IL. Knowing and outbalancing their benefits and potential flaws is indispensable for developing efficient and commercially attractive processes.

### 3.1. Purity of Ionic Liquids

The physical properties and chemical behavior of ILs, including melting point, viscosity, conductivity, polarity, reaction rates and courses, catalytic effects, and potential side reactions, can change drastically even in the presence of small amounts of impurities [93,94,95,96,97]. Residues of unreacted starting material (amines and alkyl halides), side products (inorganic salts), and, in case of non-halide ILs, compounds from the ion exchange (sodium/lithium/silver salts or acids) are often found in traces [98]. Thus, the purity of ILs has been a key issue of this type of solvents in recent years. However, compared to the situation of 5–10 years ago, the quality of commercially available ILs has been improved drastically. Advanced analytical tools for characterization of impurities have been emerged and both synthesis as well as purification techniques for ILs have been improved [99,100]. Nowadays, the majority of ILs that are frequently applied for cellulose dissolution are offered with grades of ≥98–99%, which is the same quality generally accepted for the use of common molecular solvents in organic synthesis [101,102]. However, there are some exceptions such as EMIMAc, one of the most popular cellulose solvents, which is known to be of rather low quality (90–95%). In order to make results obtained from different groups and with different ILs comparable and reproducible, full specification of the ILs employed, including purity grade, melting point, manufacturer/distributor, and batch number, should be provided in the experimental section of scientific publications. 

It is important to note that certain impurities may have an effect on cellulose derivatization. As an example; it has been demonstrated that silylation of cellulose in BMIMCl yields almost no DS when carried out in an IL of high purity (99%) while silylated products with high DS could be obtained in case of lower solvent quality (95%) [69]. The increase in reactivity could be due to traces of 1-methylimidazole that acted as catalyst for the etherification. Thus, the beneficial or adverse effect of impurities on cellulose derivatization should be considered in case unexpected results are encountered such as unusually high reactivity in ILs compared to common polysaccharide solvents.

Another important aspect is the ubiquitous presence of water that is almost unavoidable when working with ILs. Even hydrophobic ILs are known to absorb rather high amounts of moisture from air [103]. First of all, water acts as a “precipitation agent” for cellulose/IL solutions and thus influences the solubility of the polysaccharide. It has been reported that cellulose solutions in certain ILs can tolerate rather high amounts of water of up to 20 wt % [104]. Nevertheless, even traces of water impair the state of dissolution of cellulose in ILs and thus can have an adverse effect on processing or chemical modification even before the occurrence of “macroscopic precipitation”. Agglomerates of cellulose chains and formation of “micro-gels” may occur upon the addition of water. It has been demonstrated that the intrinsic viscosity of a cellulose/EMIMAc solution significantly increases with increasing water content up to a maximum around 10 wt% water and then decreases again until reaching the region were the polysaccharide becomes insoluble [105]. Intrinsic viscosity is directly correlated with the size and conformation of the dissolved polymer coil. Consequently, this phenomena can influence rheological behavior, e.g., important for fiber spinning, as well as chemical derivatization, e.g., distribution pattern.

Water can also directly influence the course of chemical derivatization reactions. Even in traces, it may lead to an apparent decrease in reactivity, due to hydrolysis of the derivatization reagent or the substituents. This effect should be considered and if need be, verified by repeating the derivatization reaction under strictly anhydrous conditions. Last but not least, the presence of water promotes degradation of the cellulose chain, especially in case of acidic reaction conditions and high temperatures [106,107]. ILs may contain residual acid derived from their synthesis, which can catalyze hydrolysis of the polysaccharide during the dissolution.

It has been noted frequently that even after intensive washing, cellulose and cellulose derivatives regenerated from ILs after the processing, *i.e.*, shaping, chemical derivatization, or pretreatment of biomass, might still contain up to several weight percent of IL residues [67,108]. In particular anionic cellulose derivatives can retain the IL’s cation and require proper purification. Moreover, ILs might chemically react with the polysaccharide (see Section 3.4.) or simply become physically entrapped upon the regeneration step. Even traces of an IL might have adverse effects on physical, chemical, and biological properties of the final cellulose product. It has been demonstrated that cellulase activity is significantly reduced when residual IL is present in the cellulosic material, which might become a drawback for the use of ILs in biomass pretreatment and biofuel production [108,109]. Relatively large amounts of IL impurities can be detected by NMR spectroscopy and elemental analysis, note that most ILs contain nitrogen or other hetero atoms. However, for the use in food- or biomedical applications more precise techniques are most likely needed.

### 3.2. Viscosity: A Matter of Kinetics vs. Thermodynamics

**Figure 3:**
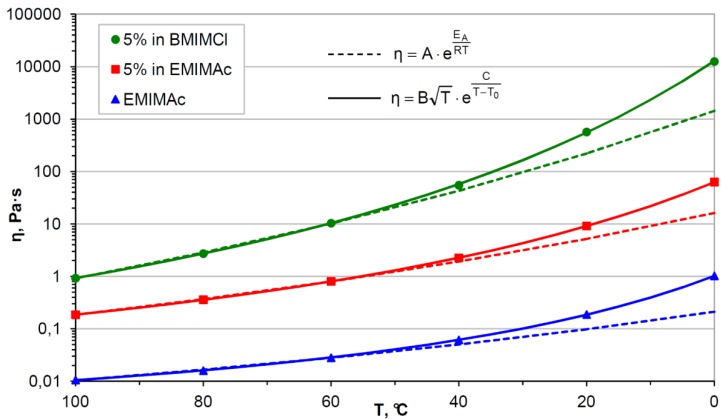
Temperature dependence of the viscosity of microcrystalline cellulose solutions in 1-ethyl-3-methylimidazolium acetate (EMIMAc) and 1-butyl-3-methylimidazolium chloride (BMIMCl). Dotted lines represent extrapolation based on the 60–100 °C-interval according to an Arrhenius equation. Straight lines representing the experimental behavior were calculated by a Vogel-Fulcher-Tamman equation, adapted from [119].

The viscosity of cellulose/IL solutions plays an important role in all steps involved in the processing of the polysaccharide from dissolution, over handling of the solutions, to the final stage, e.g., spinning of fibers or chemical derivatization in the viscous systems. Moreover, detailed knowledge on the rheological flow properties is crucial for scale up of lab procedures to commercial applications to estimate the energy input required for processing and the design of reaction vessels, stirrers, pipe systems, and other technical devices. Viscosity data for typical cellulose dissolving ILs at different temperatures are given in Table 2. It should be pointed out that impurities, in particular ubiquitous water, strongly affect the viscosity of ILs, which means that different values for one and the same IL might be found in different references. Nevertheless, it is obvious that even in case of ILs with rather low viscosity, the values are at least two orders of magnitudes higher compared to common molecular solvents. Therefore, kinetic effects have a very pronounced influence on physical- (dissolution) and chemical processes (derivatization) in cellulose/IL solutions. In contrast to many common liquids, the viscosities of ILs and their corresponding cellulose solutions shows non-Arrhenius temperature behavior and increases unexpectedly fast in particular when approaching low temperatures of 0 to 40 °C [110,111]. Compared to the values at 100 °C, the viscosity of a 5% cellulose/EMIMAc solution increases by a factor of about 100 upon cooling to 20 °C and by 1,000 at 0 °C (Figure 3). For BMIMCl, these differences are even 10-times higher.

**Table 2:** Zero-shear rate viscosities of ionic liquids, water, and dimethylsulfoxide.

	Ionic Liquid ^a^		Temperature,	Viscosity,	Ref.
	Cation	Anion	°C	mPa·s	
	AMIM^+^	Cl^−^	50	120	[112]
	AMIM^+^	Fo^−^	25	66	[113]
	BMIM^+^	Ac^−^	25	485	[114]
	BMIM^+^	Ac^−^	80	26	[114]
	BMIM^+^	Ac^−^	100	15	[114]
	BMIM^+^	Ac^−^	120	9	[114]
	BMIM^+^	Cl^−^	80	142	[114]
	BMIM^+^	Cl^−^	100	68	[114]
	BMIM^+^	Cl^−^	120	31	[114]
	BMIM^+^	Fo^−^	25	38	[112]
	BMIM^+^	DMP^−^	20	696	[115]
	BMIM^+^	DMP^−^	80	≈30	[115]
	EMIM^+^	Ac^−^	21	180	[116]
	EMIM^+^	Ac^−^	25	162	[114]
	EMIM^+^	Ac^−^	80	17	[114]
	EMIM^+^	Ac^−^	100	6	[114]
	EMIM^+^	Ac^−^	120	5	[114]
	EMIM^+^	Cl^−^	80	65	[114]
	EMIM^+^	Cl^−^	100	27	[114]
	EMIM^+^	Cl^−^	120	13	[114]
	EMIM^+^	DEP^−^	20	394	[115]
	EMIM^+^	DEP^−^	21	460	[116]
	EMIM^+^	DMP^−^	20	457	[115]
	EMIM^+^	DMP^−^	25	265	[117]
	EMIM^+^	DMP^−^	80	≈27	[115]
	water		25	0.9	[118]
	water		45	0.6	[118]
	DMSO		25	2.0	[118]
	DMSO		45	1.4	[118]

^a^ Cations: AMIM^+^: 1-allyl-3-methylimidazolium, BMIM^+^: 1-butyl-3-methylimidazolium, EMIM^+^: 1-ethyl-3-methylimidazolium, anions: Ac^−^: acetate, Cl^−^: chloride, DEP^−^: diethylposphate, DEP^−^: dimethylposphate.

The viscosity of cellulose/IL significantly affects transport properties, such as ion diffusion and mass transfer [120]. In highly viscose solutions, physical and chemical processes might be kinetically inhibited although they are thermodynamically favored and vice versa. Consequently, it is important to distinguish between kinetic and thermodynamic effects when discussing the influence of viscosity, or other parameters such as temperature and pressure, on a specific phenomenon in different ILs because the viscosity of individual ILs may easily differ by 1 or 2 orders of magnitudes. As an example; the “solubility” of cellulose in a specific IL is, according to IUPAC, defined as the “analytical composition of a saturated solution, expressed in terms of the proportion of a designated solute [cellulose] in a designated solvent [IL]” [121]. It is a thermodynamic parameter that does not depend on viscosity and can only be quantified, when solid cellulose and saturated cellulose/IL solution are in an equilibrium state. The “dissolution” of cellulose, however, is the kinetically controlled process that ultimately leads to said equilibrium. It is strongly affected by viscosity and thus might result in a pseudo-equilibrium when diffusion becomes too slow compared to the finite time frame of an experiment. In that case, an apparent solubility is obtained that is lower than the actual one. In practice, the solubility of cellulose in most cellulose dissolving ILs is very high, concentrations of 10–20% and higher have been reported, which implies that viscosities are very high as well [4,122]. Thus, it might be difficult to clearly attribute the beneficial/adverse effects that a change in external parameter, e.g., temperature, pressure, IL type, or presence of co-solvents, might have on cellulose dissolution or chemical derivatization either to a change in thermodynamics or kinetics. It has been reported that the intrinsic viscosity of cellulose/IL solutions decreases with increasing temperature, which is an indication for a decrease of the thermodynamic quality of the solvent (lower solubility) [110,111]. Nevertheless, cellulose is preferably dissolved in ILs at elevated temperatures because the decrease in viscosity favors mass transfer and enables efficient mixing (favorable dissolution). In the same way, kinetics and thermodynamics of the dissolution of cellulose in ILs are altered when the structure of the solvent is modified [123]. Viscosity of an IL and its ability to dissolve polysaccharides both depend among others on the hydrogen bond donor/acceptor properties (see Section 4.1.).

Dissolution of cellulose in ILs is a heterogeneous process that starts immediately when the polysaccharide is in contact with the solvent, which results in swelling and viscosity increase. Due to the fast dissolution it may happen that a highly viscose cellulose/IL gel is rapidly formed as skin layer that covers the surface of the cellulose bulk and prevents further dissolution. Thus, sophisticated stirring techniques are required for the efficient preparation of homogeneous cellulose/IL solutions in particular in case of high molecular weight cellulose and polymer content (15% and more). Cellulose/IL spinning dopes with high concentrations have been prepared by adopting the dissolution procedure of the NMMO process [5]. Thereby, cellulose is suspended in an aqueous IL solution and water is removed under reduced pressure and constant mixing. A higher solid-liquid interface area is obtained in this way, which kinetically favors the dissolution of high amounts of cellulose.

Analogous to the dissolution of cellulose in ILs, its chemical derivatization may be affected by the adverse effect of high viscosity. For a completely homogeneous derivatization reaction, uniform and instantaneous distribution of the derivatization reagent within the reaction mixture is required, which in practice means rapid distribution compared to the reaction time. Otherwise the reaction already yields highly substituted derivatives in direct vicinity of the reagent whereas at the same time unmodified cellulose is still present in other parts of the reaction vessel. These effects are particularly pronounced for reactions performed at or below room temperature. Derivatives with a small DS but a uniform distribution pattern are difficult to obtain.

Acylation of cellulose in ILs has been found to be very efficient. It is performed at elevated temperature, *i.e.*, low viscosity, and with the use of liquid derivatization reagents. Thus, efficient mixing is guaranteed and uniform products could be obtained easily. In contrast, derivatization reactions that have to be carried out at low temperature have been demonstrated to be more difficult when performed in ILs. Tosylation of cellulose is commonly performed at or below room temperature in order to avoid S_N_ of the tosyl moiety formed with chloride ions (formation of chloro-deoxycellulose) or hydroxyl groups of the polysaccharide (formation of cross-linked, insoluble derivatives) [124]. However, conversion of cellulose with tosyl chloride in ILs at 10 °C has been found to yield a product mixture of tosylated derivative (65%, DS_tosyl_ ≈ 1) and unmodified cellulose (45%, DS_tosyl_ ≈ 0) due to the insufficient mixing during the reaction [74]. Inhomogeneous derivatization and only partially soluble products have also been observed for the preparation of cellulose sulfate in ILs at 25 °C [71]. In contrast, sulfation at 60–80 °C yielded completely soluble but strongly degraded products due to the rather acidic reaction conditions. For these and other derivatization reactions, co-solvents have been applied to diminish the viscosity of cellulose/IL solution and thus enable completely homogeneous derivatization (see Section 4.2.).

### 3.3. Hydrophobic Reagents in Hydrophilic Solvents—Homogeneous or Heterogeneous Derivatization

Although cellulose itself is soluble in ILs, it has to be emphasized that the chemical modifications of the polysaccharide in these media are not necessarily proceeding under homogeneous reaction conditions. So far, all ILs that have been exploited for dissolution of cellulose are relatively hydrophilic (see Section 4.1.). As a consequence, addition of hydrophobic bases, reagents, or co-solvents might result in formation of a separate phase and consequently a heterogeneous reaction course. It has been demonstrated that qualitative predictions on miscibility with ILs are possible based on the solvatochromic parameters of a compound [125]. 

Derivatization of cellulose in ILs may start homogeneously but results in phase separation when the derivative formed becomes increasingly hydrophobic upon increasing DS. Although it is not necessarily the case, precipitation of the hydrophobic derivative from the reaction mixture may decrease reactivity towards further substitution as has been demonstrated for the conversion of cellulose, dissolved in ILs, with lauroyl chloride that could not yield products with DS > 1.5 [36]. Precipitation from the IL reaction medium has also been described for trimethylsilyl cellulose with a DS > 2 [69]. In addition, the silylation reagent used is not miscible with cellulose/IL solutions. Completely homogeneous silylation up to a DS of 2.9 could be performed with the aid of chloroform as co-solvent [69]. On the other hand, heterogeneous silylation of cellulose in biphasic IL/co-solvent systems has been performed using toluene, which only dissolves the reagent and highly substituted cellulose silyl ethers [70]. It is reasonable to assume that different reaction courses will also yield products that differ in their properties. Certain inorganic compounds such as NaOH, utilized as base for the carboxymethylation of cellulose, and iodine, used as catalyst for the acylation, are not soluble in ILs as well [41,52].

The heterogeneous conversion of cellulose in ILs with IL-immiscible compounds strongly dependents on transition of reagents and products from one phase into the other, which is influenced, among others by temperature, viscosity, stirring speed, and area of the liquid/liquid interface. These parameters will significantly affect the properties of the products obtained. With increasing DS, solubility of the cellulose derivatives obtained will change and substituted molecules, formed in direct vicinity of the liquid/liquid interface fill diffuse into the non-polar phase, whereas unmodified cellulose remains dissolved in the IL. Since the reaction velocity in both phases is most likely not the same, further derivatization of already modified cellulose molecules may proceed differently compared to non-substituted ones. Thus, heterogeneous derivatization of cellulose is much more prone to yield non-uniform product mixtures or derivatives with an uneven distribution pattern. Since ILs offer the possibility for homogeneous cellulose modification, it appears to be reasonable to either substitute IL-immiscible compounds or to improve their miscibility, e.g., by designing task-specific IL or utilizing co-solvents.

### 3.4. Ionic Liquids as Non-Innocent Solvents—Thermal Stability and Side Reactions

**Figure 4:**
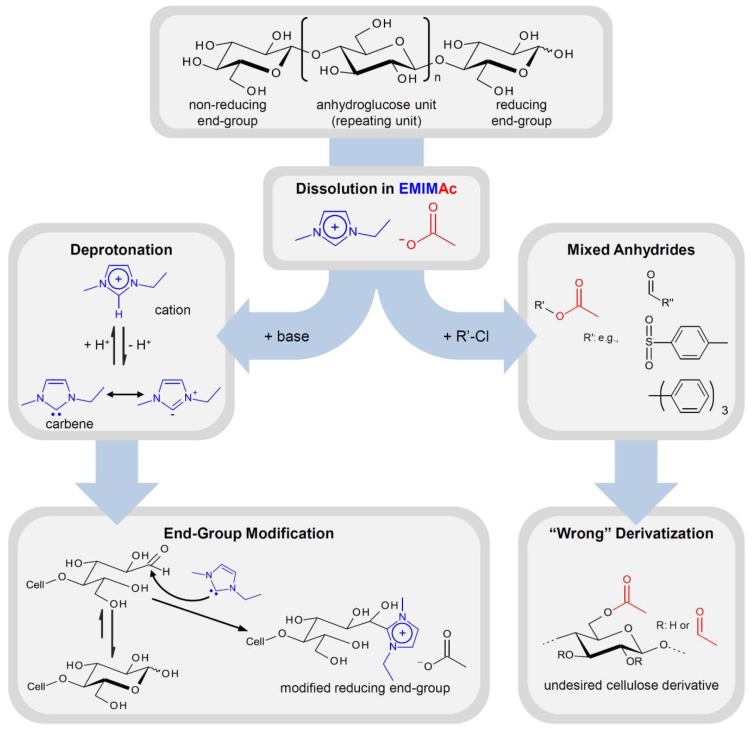
Schematic representation of side reactions, observed upon the dissolution and chemical derivatization of cellulose in 1-ethyl-3-methylimidazolium acetate (EMIMAc).

In particular imidazolium-based ILs, which are the most frequently ones applied in cellulose chemistry, are “non-innocent solvents” meaning that they are not necessarily chemically inert. They can participate in the derivatization reaction, alter the reactivity, or induce the formation of unexpected products [126,127]. In 1,3-dialkylimidazolium salts, the proton at C-2 is rather acidic with a p*K*_a_ of about 21–24, determined in DMSO and water [128,129]. Deprotonation yields singlet *N*-heterocyclic carbenes that are stabilized by the two adjacent nitrogen atoms. These reactive nucleophilic species occur as intermediates in the catalytic cycles of many organic syntheses that have been performed in ILs with surprisingly high yields or with unexpected reaction products [130,131]. It has to be considered that also derivatization of cellulose in ILs might be affected by the presences of carbenes in particular when bases are applied (Figure 4).

Due to the comparably high basicity of their anion, imidazolium acetate-based ILs may undergo self-deprotonation, even in the absence of additional base [132,133]. The carbene species formed might be present only in small concentrations but they can react with the reducing end group of cellulose in its aldehyde form, shifting the equilibrium further to the product side (Figure 4). This effect has been described first for water insoluble cellodextrin (DP = 7), dissolved in ILs [3]. Later on, it has been demonstrated for ^13^C labeled glucose that 15–20% of the reducing glucopyranose are converted after storage for one week in an imidazolium acetate [134]. In the presence of an additional base the same result was obtained after 2 h. The effect of the reducing end group may diminish with increasing DP of the cellulose chain but it should not be dismissed. In particular studies aimed to elucidate the mechanism of dissolution of cellulose in EMIMAc have to consider these effects that might lead to miss interpretation of experimental data when glucose or cellobiose are applied as cellulose analogues (see Section 4.1.). Imidazolium salts, methylated at C-2, have been proposed frequently as cellulose solvents in order to avoid the adverse effect of intermediate carbenes [36]. However, also the methyl group can be deprotonated to a certain extent, which induces unexpected side reactions as well [135].

One of the most frequently quoted properties of ILs in general is their remarkably high liquid range and the fact that they withstand even temperatures of up to 400 °C [13]. Long-term thermogravimetric measurements at slow heating rates, however, demonstrated that under practical conditions decomposition may occur at much lower temperatures, in particular in the presence of impurities [136]. The thermal behavior of some ILs and their corresponding cellulose solutions has been studied by thermogravimetry as well as differential scanning- and reaction calorimetry [137,138]. Onset temperatures (T_on_) for the chemical decomposition and liberation of gaseous compounds around 180–220 °C have been observed and the values changed only slightly upon the addition of additives such as silver or activated charcoal particles. For comparison; cellulose/NMMO solutions are significantly less stable (T_on_ ≈ 130–160) and require the addition of stabilizers in order to prevent autocatalytic thermal runaway reactions [139,140]. The efforts required for safe handling are a major drawback of NMMO, which is applied commercially for the fabrication of cellulosic Lyocell fibers. ILs appear to be safe for use since their T_on_ lie above the processing temperature for dissolution, fiber spinning, and chemical derivatization of cellulose, which are usually below 130 °C. However, it has been observed that thermostability of cellulose/IL solutions significantly decreases when using recycled ILs instead of fresh ones, which indicates that partial decomposition may start already below the cited temperatures [137,138]. These findings are of huge importance for the use of ILs as cellulose solvents in large scale applications. IL recycling is indispensible in order to make the processes profitable (see Section 3.6.), which implies that the solvent will undergo several heating cycles.

Thermal decomposition of imidazolium-based ILs proceeds inversely to their synthesis by dealkylation yielding 1-alkylimidazoles [141,142]. The rate of this reaction depends on nucleophilicity and size of the anion as well as the length of the alkyl chain. Freshly purified ethyl- and butyl-imidazolium salts with chloride and acetate counterions that were heated under nitrogen for 24 h at 200 °C have been found to contain around 0.01 to 0.001% degradation products, mainly 1-alkyl-imidazoles, imidazole, and dimerization derivatives therefrom [143]. Due to the high basicity, even these small amounts can significantly affect dissolution and especially chemical derivatization of cellulose in ILs. Moreover, these compounds could not be removed from ILs simply by evaporation. Consequently, they will accumulate in the IL upon multiple recycling. 

The IL’s anion, which is present in very high concentrations and not shielded by a cage of organic solvent molecules, can undergo side reactions as well. It has been reported that conversion of cellulose, dissolved in EMIMAc, with furoyl-, tosyl-, and trityl chloride as well as SO_3_-complexes yields cellulose acetate in all cases [144]. Conversion of acetate with the derivatization reagents and formation of mixed anhydrides that ultimately act as acetylation reagent has been demonstrated by means of NMR spectroscopy (Figure 4). On the contrary, performing the same derivatization reactions in BMIMCl gives the expected derivatives (see Table 1, entries **6**, **22**, **18**, **21**) [47,67,74,119]. It has been noted that acetylation of cellulose in EMIMAc takes place, to a very low extent, also in the absence of additional derivatization reagents [145]. It should be pointed out that unexpected side reactions in ILs are not necessarily a drawback. If well understood they might be exploited for the preparation of well-defined cellulose derivatives. Many types of reactions as well as catalyst that are commonly utilized nowadays were developed from results that were unexpected or undesired in the very beginning. It has been found that the acetate anion has a beneficial effect for hydroxyalkylation of cellulose in ILs by acting as a catalyst in the ring opening reaction [69].

### 3.5. Toxicity and “Greenness” of Ionic Liquids

ILs have attracted increasing interest not only in the area of polysaccharide research but also as novel solvents and reaction media in electrochemistry, analytical chemistry, and organic synthesis in general [13,29,30,31]. In particular the need to replace volatile organic solvents in these processes by preferably less harmful solvents with low vapor pressure is one of the driving forces for the rapid developments in the field of IL research. The potential use of ILs in commercial processes makes the evaluation of their hazardous potential for man and environment very important [146,147]. Like all of their properties, toxicity of ILs strongly depends on the nature of cation and anion and thus no general statements can be advanced [148,149,150]. If a specific IL is “safe to use” has to be decided individually for the specific process. To give just one example; BMIMCl, which is one of the most frequently applied ILs for cellulose dissolution, is listed as “toxic if swallowed” with LD_50 rat/oral_ of 50–300 mg/kg, which is comparable to caffeine and acetylsalicylic acid [151]. Cellulose dissolving ILs are relatively hydrophilic and possess low octanol-water partition coefficients, which suggests that they will not bioaccumulate to high extent in aqueous organisms [152]. Nonetheless, with the risk of pollution from industrial processes, biodegradability of ILs in aquatic and soil environment is an important issue [153,154].

The question if and to what extent ILs are truly “green solvents” is of significant general concern and discussed intensively [155,156]. For a comprehensive evaluation, different aspects have to be considered starting from synthesis of the solvent, over its properties, e.g., toxicity, volatility, risk of exposure, to the final recycling or disposure. Thus, the “greenness” strongly depends on the particular IL and the process in which it is supposed to be advantageous over other solvents. None of the numerous cellulose solvents known in literature is currently applied for the preparation of cellulose derivatives in considerable commercial scales. Consequently, the greenness of ILs appears to be less relevant for their use as reaction media for derivatization of cellulose. After all, ILs are intensively studied in this area because they are, unlike other solvents that might even be “greener”, able to dissolve cellulose and facilitate a vast number of chemical derivatization reactions. Regarding, the production of cellulosic fibers, ILs have to compete with other cellulose solvents already established decades ago. Whether or not IL-based procedures are attractive alternatives to the currently applied viscose- or the NMMO-based Lyocell process is still a matter of ongoing research [157]. Careful evaluation of ecological and economical aspects is also required when ILs are aimed to be used in the production of biofuel and platform chemicals from lignocellulosic biomass [122]. In this context, the requirement for efficient removal of IL traces from the cellulosic materials should be pointed out again (see Section 3.1.).

### 3.6. Recyclability of Ionic Liquids

Taking into account the rather high costs of ILs and the increasing concern for environmental and safety issues (see also Section 3.5.), efficient recycling of ILs is one of the key issues that need to be solved in order to apply these solvents in industrial scale processes that are profitable and sustainable [13]. Otherwise, ILs will share the fate of other cellulose solvents that are only used in academic research such as DMA/LiCl that proved to be an efficient reaction medium for the derivatization of cellulose in lab-scales but never found use in industrial applications due to its high cost and limited possibility to reuse the solvent [33,34,35]. Recycling of LiCl from the reaction is difficult while separation and purification of DMA by evaporation is costly and energy consuming due to its low volatility.

If ILs are used as solvents for the fabrication of cellulosic fibers they have to compete with the viscose- and Lyocell process that are well established commercialized practices. In the later case, 99% of the cellulose solvent NMMO are recovered implying that comparable rates are required for ILs in order to make them economically attractive [158]. Depending on the fixed costs of the derivatization reagent, lower recycling rates might be sufficient for a commercialized process using ILs as reaction media for the derivatization of cellulose.

**Figure 5:**
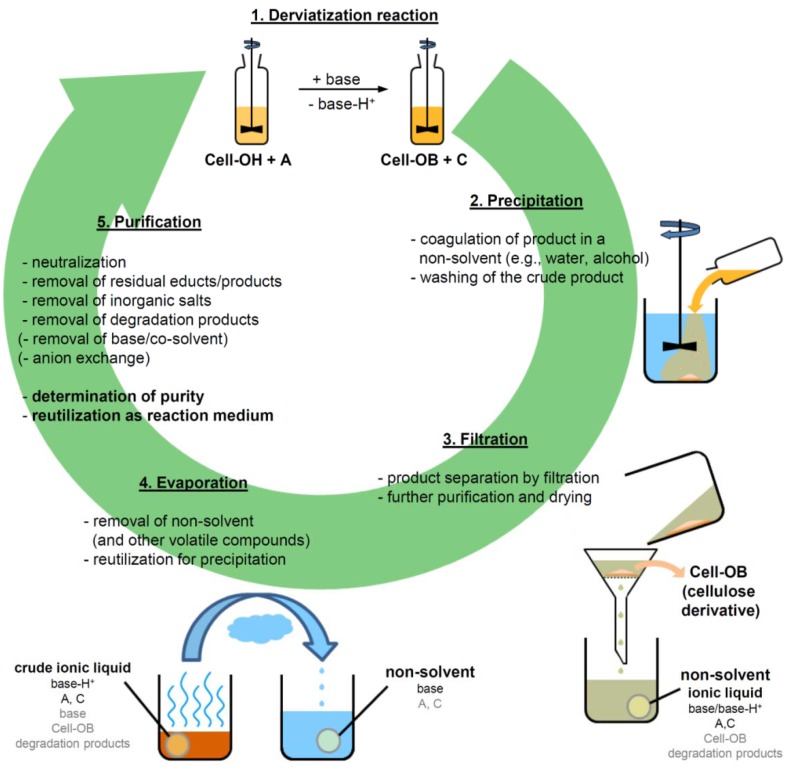
Scheme for the derivatization of cellulose in an ionic liquid including product isolation and solvent recycling.

As already pointed out, impurities from the derivatization reaction and from thermal decomposition of the ILs may accumulate over several recycling cycles and have to be removed, not necessarily completely but to an extent where they do not influence dissolution and chemical derivatization of cellulose. A typical derivatization reaction of cellulose in an IL is schematically depicted in Figure 5. Subsequent to the chemical synthesis and isolation of the cellulose derivative by precipitation in a non-solvent, a filtrate is obtained that contains the IL, together with side products, not consumed starting materials, and the base, if utilized. Although evaporation of the non-solvent and volatile impurities might be an energy intensive procedure, depending on the boiling points of the individual compounds, it yields a crude IL that, in a few cases, could be utilized directly for a second derivatization. In general, further purification is required. In the absence of bases, acylation of cellulose in imidazolium chloride-based ILs yields hydrochloric- or carboxylic acids as side products that are easily removed together with the volatile non-solvent used for precipitation and the excess acylation reagents. According to their NMR spectra, the recycled ILs contained no impurities and when reused as reaction media, the results were comparable to fresh ILs [40,41,46]. ILs, utilized for the homogeneous silylation of cellulose with hexamethyldisilazane could be recycled in a comparable way due to the low boiling points of the side products formed, e.g., ammonia and silazanes [69]. In contrast, acylation of cellulose in imidazolium acetate with the use of carboxylic acid chlorides will most like lead to a partial ion exchange to imidazolium chlorides. Due to the higher basicity of carboxylic acids, these compounds will evaporate first. A complex mixture of recycled ILs can also be expected when mixed cellulose esters are prepared [45].

The recycling of ILs proved to be much more difficult in the presence of bases, applied to aid the derivatization of cellulose, e.g., in tosylation (entry **22**) and tritylation (entry **18**), or when non-volatile side products are formed [67,74]. Although the base itself will be removed in most cases upon evaporation of the precipitating agent and other volatile compounds, the corresponding protonated acid will remain dissolved in the crude IL. For comparison; the boiling points of pyridine and pyridinium hydrochloride are 115 °C and 223 °C. In order to prevent cellulose degradation during dissolution in recycled ILs, complete removal of these acidic compounds is required, which could be achieved by neutralization in aqueous media and removal of water and the deprotonated base under reduced pressure [71]. Finally, the crude product is extracted with chloroform to separate the soluble IL from insoluble inorganic salts, derived from the derivatization and neutralization, which precipitate and can be remove by filtration. Bases with a high boiling point and sufficiently low polarity can be removed from the neutralized aqueous IL solution by extraction with a non-polar solvent. 1-Butyl- and 1-benzylimidazole have been applied for homogeneous tosylation of cellulose in mixtures of IL and a co-solvent and could be removed according to said recycling procedure [74]. Both compounds possess basicities comparable to imidazole and 1-methylimidazole but are less hydrophilic, which results in an increased partition coefficient in the non-polar phase and favorable extraction [159]. Derivatization might involve the formation of anionic species that cannot be removed efficiently by the procedures described because the corresponding protonated acid is not volatile, e.g., tosylate, or because the properties of the novel anion formed are too similar to those of the original one, e.g., acetate *vs.* propionate. In these cases, treatment of the crude IL with an anion exchange resin might be necessary. 

Addition of ILs to concentrated aqueous solutions of water-structuring salts, e.g., phosphates, carbonates, citrates, or certain organic compounds, e.g., carbohydrates, amino acids, surfactants, polymers, has been found to induce the formation of two separate phases, one of them IL-rich, the other IL-deficient [160,161]. Salting-out has been applied for purification and recovery of AMIMCl used for acylation of cellulose [42]. The IL could be recycled with 85% yield and, according to its ^1^H-NMR spectrum, contained no organic impurities. However, no information on residual inorganic salts were provided. Little attention has been paid so far on the effect of inorganic impurities that may derive from derivatization, subsequent recycling, or from the cellulosic raw material itself, the chemicals used in the pulping process, and metal devices used in any of the processing steps. In particular metal ions such as copper or iron can have significant influence on chemical and thermal properties of ILs and on the biocompatibility of cellulose derivatives and materials processed therefrom [162]. The presence of metal traces also influences yield and product composition of the acid catalyzed hydrolysis of cellulose in ILs [163]. Consequently, monitoring the concentration of metal ions over several recycling cycles can become important in particular when they are required for the derivatization of cellulose in ILs, e.g., as catalyst for ATRP. Inorganic compounds are usually not detectable by ^1^H- or ^13^C-NMR spectroscopy, which have been up to now the standard techniques to verify the degree of purity of recycled IL. For the use of ILs as cellulose solvent in large scale applications (fiber spinning, biorefinery, preparation of bulk derivatives) additional characterization methods such as ion chromatography might be useful [164].

In addition to salting-out, other techniques have been discussed in particular for recycling of ILs used for cellulosic fiber production: pervaporation, nanofiltration, reverse osmosis, and utilization of “distillable ILs” [165,166,167,168]. Further studies are required to clarify if these procedures are feasible, profitable in commercially scales, and can be applied to remove impurities derived from derivatization reactions performed in ILs. 

## 4. Current Developments and Future Perspectives

Despite the fact that ILs are challenging compounds to work with, they offer great potential as reaction media for the homogeneous derivatization of cellulose. After realizing potential bottle necks for the use of ILs in polysaccharide chemistry, current research is strongly focused on overcoming these drawbacks by gaining more insight in IL/cellulose interactions, developing efficient recycling strategies, and finding ways to diminish the intrinsic limitations of ILs regarding their physical and chemical properties, e.g., viscosity, melting point, hydrophilicity. Ultimately, these efforts will enable the efficient use of ILs as cellulose solvents for various processes, known and novel ones.

### 4.1. Elucidation of the Dissolution Mechanism

Understanding the interactions between ILs and cellulose is of great scientific importance and the crucial issue for developing novel, task specific ILs. A lot of effort has been put into the elucidation of the dissolution mechanism by using different techniques including, NMR spectroscopy and computational simulations (Table 3). By means of ^13^C-NMR spectroscopy, it has been demonstrated that ILs are non-derivatizing solvents, *i.e.*, dissolution is not result of any chemical derivatization but of physical solvent-solute interactions [41]. Despite that well accepted fact, no general theory exists that fully describes the dissolution of cellulose in ILs. The individual contribution of the IL’s anion and cation to the dissolution mechanism as well as the question if only one of them or both interact with the polysaccharide backbone is a matter of controversial debates [169,170,171,172]. Nevertheless, both species (directly or indirectly) influence the dissolution of cellulose in ILs. Completely separated consideration of anion and cation is not pertinent; if nothing else because both are inherent components of an IL that determine its physical properties, e.g., melting point, viscosity, density. As described above (Figure 2), cellulose dissolving ILs are restricted in the type of anion, which implies its importance for the dissolution. On the other hand, the influence of the cation must not be neglected. As an example; it has been demonstrated that the solubility of cellulose in molten 1-alkyl-3-methylimdazlium chlorides depends on the length of the alkyl chain [173]. Moreover, ILs with an odd number of carbon atoms in the alkyl chain have been described to dissolved only small amounts of cellulose compared to ones with an even number. This odd/even phenomenon that indirectly influences cellulose dissolution was ascribed to an ordered/disordered packing of the cations.

Critical discussion on an interdisciplinary level has always been the basis for solving scientific “miracles”. Instead of analyzing and judging numerous partly contradicting studies on the complex dissolution mechanism from a single point of view, the authors would like to promote discussions by providing references of significance (Table 3) and encouraging readers with different background and expertise to participate.

**Table 3:** Experiments performed to elucidate the mechanism of cellulose dissolution in ionic liquids.

Experimental Technique	Model Compound	Ref.
computational simulation	glucose, cellodextrins (DP = 2–12)	[174]
computational simulation	glucose	[175,176]
computational simulation	cellobiose	[177]
computational simulation	cellodextrins (DP = 5–20)	[178]
computational simulation	(1,4)-dimethoxy-β-D-glucopyranose	[179]
computational simulation	cellulose microfibrils (36 glucan chains, DP = 16)	[180,181]
computational simulation	cellodextrin (DP = 20)	[182]
computational simulation	cellulose microfibrils (36 glucan chains, DP = 20)	[183]
computational simulation	cellulose Iβ crystal	[184]
computational simulation	cellobiose	[185]
computational simulation	(1,4)-dimethoxy-β-D-glucopyranose	[186]
IR spectroscopy(computational simulation)	pentaerythritol	[187]
neutron diffraction(computational simulation)(NMR spectroscopy)	glucose	[188]
NMR spectroscopy	glucose, cellobiose	[169,189,190]
NMR spectroscopy	cellobiose	[170]
NMR spectroscopy(solvatochromic parameters)	ethanol	[191]
solvatochromic parameters	cellulose	[192]
solvatochromic parameters	cellulose	[193]
solvatochromic parameters	cellulose	[194]
X-ray diffraction	cellulose	[195]

From the perspective of a polysaccharide chemist it has to be noted that extrapolation of results obtained from glucose and cellobiose, which have been utilized as model compounds in most of the cited studies, to the polymer cellulose has to be done with caution. Cellulose is composed of several hundred *β*-(1–4) linked AGU flanked by a reducing- and a non-reducing end-group (see Figure 4). Later have almost no impact on the overall properties of the polysaccharide. Strictly speaking, celltriose is the first model compound that contains all structural features of cellulose whereas glucose and cellobiose only mimic the two end-groups. It was already described that the reducing end groups of cellulose, in its aldehyde form, may react with ILs, which might result in misleading interpretations of experimental data [3,134]. Interactions of ILs with the end-groups play no role in the dissolution process. Thus, short chain non-reducing *β*-D-cellulosides with a DP of 15–20 that lack the ability to undergo these misleading interactions are more suitable model compounds for the polysaccharide [196]. Like cellulose, these substances are insoluble in water and organic solvents, which is a feature not shared by glucose and cellobiose. Due to the low DP, the viscosity of cellulosides/IL solutions is sufficiently low to avoid interference with the experiments. 

It has been demonstrated that the IL’s hydrogen bond acceptor ability is an important aspect that determines whether or not cellulose can be dissolved [191]. A more qualitative and phenomenological approach to understand dissolution of cellulose is therefore based on the comparison of empirical solvatochromic parameters of different ILs that are or are not a cellulose solvent (Table 4) [192,193,194]. 

Precise quantitative statements are difficult because literature values for solvatochromic parameters of ILs may differ between different studies, due to the use of different techniques, probe molecules, concentrations, and the strong influence of impurities. Nevertheless, it can be stated that only ILs that are strong hydrogen bond acceptors (*β* > 0.8) are able to dissolve cellulose. The *β*-value is mainly determined by the nature of the IL’s anions, which confirms the assumption that it plays a crucial role in the dissolution mechanism. In order to exploit this qualitative approach, e.g., for the synthesis of task specific ILs, further studies are required to clarify the significance of the other parameters (*E*_T_^N^, *α*,π^*^) in correlation to the molecular structure of the cation. A suitable approach to study these questions is to dilute ILs with a co-solvent or a non-solvent and to study dissolution of cellulose in these mixtures with respect to the change of the solvatochromic properties [194,197].

**Table 4:** Solvatochromic parameters of ionic liquids that are or are not able to dissolve cellulose.

	Ionic liquid ^a^			Solvatochromic parameters ^b^					Dissolves cellulose	Ref.
	Cation	Anion		*E*_T_^N^	*α*	*β*	*π* ^*^			
	AMIM^+^	Fo^−^		n.a.	0.48	0.99	1.08		yes	[198]
	AMIM^+^	MPo^−^		n.a.	0.51	0.99	1.06		yes	[123]
	BMIM^+^	Ac^−^		0.611	0.43	1.05	1.04		yes	[199]
	BMIM^+^	Ac^−^		0.892	0.57	0.99	0.97		yes	[44]
	BMIM^+^	Ac^−^		n.a.	0.55	1.09	0.99		yes	[113]
	BMIM^+^	Ac^−^		n.a.	0.43	1.20	n.a.		yes	[193]
	BMIM^+^	Ac^−^		n.a.	0.36	0.85	n.a.		yes	[200]
	BMIM^+^	Ac^−^		n.a.	n.a.	1.16	n.a.		yes	[192]
	BMIM^+^	Cl^−^		0.901	0.51	0.84	1.08		yes	[44]
	BMIM^+^	Cl^−^		n.a.	0.47	0.87	1.10		yes	[113]
	BMIM^+^	Cl^−^		n.a.	0.49	0.83	1.03		yes	[193]
	BMIM^+^	Fo^−^		n.a.	0.56	1.01	1.03		yes	[113]
	BMIM^+^	MPo^−^		n.a.	0.52	1.02	1.01		yes	[123]
	EDMIM^+^	MPo^−^		n.a.	0.33	1.01	1.11		yes	[123]
	EMIM^+^	DMP^−^		n.a.	0.51	1.0	1.06		yes	[117]
	EMIM^+^	MPo^−^		n.a.	0.52	1.0	1.06		yes	[123]
	EMPIP^+^	MPo^−^		n.a.	0.29	1.08	1.08		yes	[123]
	HEMIM^+^	MPo^−^		n.a.	0.63	0.91	1.06		yes	[123]
	MOEMIM^+^	Ac^−^		0.912	0.59	1.06	1.01		yes	[44]
	MOEMIM^+^	MPo^−^		n.a.	0.51	0.98	1.07		yes	[123]
	TEMA^+^	MPo^−^		n.a.	0.29	1.04	1.14		yes	[123]
	BMIM^+^	CH_3_SO_4_^−^		n.a.	0.54	0.67	1.05		no	[193]
	BMIM^+^	N(CN)_2_^−^		n.a.	0.44	0.64	n.a.		no	[193]
	BMIM^+^	BF_4_^−^		0.670	0.63	0.38	1.05		no	[199]
	BMIM^+^	TfO^−^		0.630	0.62	0.46	1.0		no	[199]
	Molecular solvents									
	methanol			0.762	0.98	0.66	0.60		no	[201]
	DMSO			0.444	0.0	0.76	1.00		no	[201]
	Molecular solvents (*cont.*)									
	pyridine			0.302	0.0	0.64	0.87		no	[201]
	chloroform			0.259	0.20	0.10	0.58		no	[201]
	toluene			0.099	0.0	0.11	0.54		no	[201]
	hexane			0.009	0.0	0.0	−0.40		no	[201]

^a^ cations: ABIM^+^: 1-allyl-3-butylimidazolium, ADMIM^+^: 1-allyl-2,3-dimethylimidazolium, AMIM^+^: 1-allyl-3-methylimidazolium, BDMIM^+^: 1-butyl-2,3-dimethylimidazolium, BMIM^+^: 1-butyl-3-methylimidazolium, BDMTA^+^: benzyldimethyltetradecylammonium, DOHMIM^+^: 1-(3,6-dioxa-(1-heptyl))-3-methylimidazolium, EDMIM^+^: 1-ethyl-2,3-dimethylimidazolium, EMIM^+^: 1-ethyl-3-methylimidazolium, EMPIP^+^: 1-ethyl-1-methylpiperidinium, HEMIM^+^: 1-hydroxyethyl-3-methylimidazolium, HMIM^+^: 1-hexyl-3-methylimidazolium, MOEMIM^+^: 1-(2-methoxyethyl)-3-methylimidazolium, TBMA^+^: tributylmethylammonium, TEMA^+^: triethtylmethylammonium, anions: Ac^−^: acetate, Br^−^: bromide, Bz^−^: benzoate, Cl^−^: chloride, DEP^−^: diethylposphate, DEP^−^: dimethylposphate, Fo^−^: formate, MPo^−^: methylphosphonate, Pr^−^: propionate, TfO^−^: trifluoromethanesulfonate; ^b^*E*_T_^N^: normalized empirical polarity; *α*: hydrogen bond donor ability; *β*: hydrogen bond acceptors ability; π^*^: dipolarity/polarizability.

### 4.2. Ionic Liquid/Co-Solvent Mixtures as Tailored Reaction Media

The adverse effect of high viscosity of cellulose/IL solutions and their limited miscibility with hydrophobic or solid compounds can be omitted by adding co-solvents to the reaction media. Although not in any case explicitly mentioned, many of the homogeneous derivatization reactions discussed above have been performed not in pure ILs but in IL/co-solvent mixtures (see Table 1). Even in patented procedures of commercial interest, the beneficial use of co-solvents has been described [20,56,65,72,75]. The use of chloroform as non-polar co-solvent for trimethylsilylation of cellulose in ILs has already been described (see 3.3). For dipolar aprotic co-solvents (DMSO, DMF) it has been demonstrated that the viscosity of cellulose/IL solutions decreases exponentially with the amount of co-solvent added [65]. Moreover, rheological studies have indicated that the conformation of cellulose dissolved in ILs is not impaired by the addition of DMSO [202].

Homogeneous preparation of cellulose sulfates, tosylates, and hydroxyalkyl ethers could be performed in IL/co-solvent mixtures [64,71,74]. Thereby, the dipolar aprotic co-solvents enabled mixing and rapid dissolution of the solid (sulfation, tosylation) or gaseous (alkylation) reagents in the otherwise too viscose IL reaction media. Pyridine has been reported to reduce the viscosity of cellulose/IL solutions as well [116]. Thus, it has been applied in slight excess for tosylation and tritylation of cellulose in dual nature: as a base that promotes the derivatization reaction and likewise as a co-solvent that guarantees efficient miscibility of the reaction medium [66,74]. Co-solvents can also be exploited as derivatization reagents. Novel amino cellulose esters (Table 1, entry **12**) have been prepared in BMIMCl by using *N*-methyl-2-pyrolidone and comparable lactames as co-solvents [55,56]. Upon activation with tosyl chloride, the cyclic amides reacted as esterification agents with hydroxyl groups of the polysaccharide to give an intermediate that rearranges under ring opening to yield the said products.

Any additional compound (co-solvents, bases, reagents) that is utilized for the homogeneous derivatization of cellulose in ILs has to be miscible with the reaction medium without inducing precipitation. General guidelines have been proposed that enable semi-quantitative prediction of these phenomena based on solvatochromic parameters of a compound [125]. As a rule of thumb, co-solvents with low hydrogen bond donor acidity (*α* < 0.5) and high ‑basicity (*β* > 0.4) can be added in reasonable amounts without resulting in precipitation. In addition, only compounds with high normalized empirical polarity (*E*_T_^N^) > 0.3 are miscible with cellulose/IL solutions. Self-evidently, co-solvents need to be incorporated into the recycling process. Co-solvents with a low boiling point, e.g., pyridine and chloroform, can be removed by fractionated evaporation and reused together with the purified IL. Furthermore, it has been demonstrated that cellulose can be dissolved directly in mixtures of ILs with comparablely high amounts of co-solvents [194]. In particular dipolar aprotic ones, which are known to possess high boiling points, are tolerated to a high extent. Thus, it is only necessary to remove impurities that may interfere with the derivatization reaction. The purified IL/co-solvent mixtures can then be reused for dissolution and derivatization of cellulose. In addition to classical organic compounds, supercritical CO_2_ has been proposed as co-solvent for cellulose/IL solutions [203].

### 4.3. Task Specific Cellulose Solvents for Dissolution, Shaping, and Chemical Modification of Cellulose

By combining the various cations and anion displayed in Figure 2, over 300 ILs that are likely to dissolve cellulose can be obtained. Nevertheless, the vast majority of studies on the use of ILs for shaping, biorefinery, and chemical derivatization of cellulose have been performed using BMIMCl, AMIMCl, EMIMAc, or ILs that slightly differed in the length of their alkyl chain. It is obvious that the structural diversity of ILs, which is one of the main features of this solvent class, is far from being fully exploited in polysaccharide research. Thus, introducing task specific ILs offers huge potential for the future. On the one hand, novel ILs can be synthesized based on the advanced knowledge gained. Thereby, the main goal is to obtain ILs that can dissolve cellulose but possess lower melting points and viscosities then currently applied ones. Synthesis of less hydrophilic ILs is another promising task. It is not reasonable to assume that one particular IL is suitable for all kinds of derivatization reactions. Thus, using task-specific ILs also means to simply choose the most suitable IL for a particular reaction from those that are available based on questions such as: What side reactions may occur between the particular IL, cellulose, and the derivatization reagents? Is the derivatization reaction particularly sensitive to high viscosities or involves hydrophobic reagents? What aspects have to be considered regarding the recycling process?

Synthesis of novel ILs involves four parts: (i) design of the cation, (ii) selection of the anion, (iii) combination of cation and anion to an IL, (iv) characterization of IL properties including the ability to dissolve cellulose. Knowledge on the parameters that determine the dissolution mechanism and on how these can be tailored by advanced organic synthesis is a key issue and an interdisciplinary task. High batch screening may help to evaluate the dissolution capacities of new ILs in order to extract potential candidates for deeper studies [84].

Imidazolium-based ILs are by far the most frequently applied ones in cellulose research but they show some specific side reactions and are comparably expensive. Quaternary ammonium salts, prepared by stepwise alkylation of primary, secondary, or tertiary amines, exhibit a broad structural diversity due to the fact that four alkyl chains can be variegated easily, compared to two in case of imidazolium salts. However, a certain degree of asymmetry appears to be required in order to obtain low melting points [204]. A series of alkoxy- and dialkoxy-functionalized ammonium acetates with melting points around 30–40 °C has been prepared recently [205]. Their ability to dissolve cellulose was comparable to EMIMAc although they showed slightly higher melting points and viscosities. Studying chemical derivatization of cellulose in these ILs might be interesting with respect to the side reaction that may occur. Triethylmethyl- and tributylmethylammonium formates have been prepared by conversion of the corresponding methyl carbonates with formic acid. Regarding the strict definition provided in the beginning of this review, these salts are not ILs since their melting points were above 100 °C [206]. In the presence of small amounts of formic acid, however, the salts melt around 80 °C and dissolve cellulose without polymer degradation. Pyrolidinium-, piperidinium-, and morpholinium-based ILs, derived from the cyclic amines, have been proposed as solvents for dissolution and processing of cellulose and lignocellulosic materials [123,207]. Morpholinium salts are particularly interesting from a scientific point of view because of their kinship with NMMO and the question if conclusions on the dissolution mechanism in both solvents can be drawn from their structural resemblance. ILs derived from 1,8-diazabicyclo[5.4.0]undec-7-ene (DBU), which is known as a strong, non-nucleophilic base, have been described by BASF for dissolution of cellulose and other polymeric compounds [208]. Guanidinium carboxylates with melting points between 60 and 100 °C have been applied successfully for cellulose dissolution [168]. These ILs decompose to tetramethylguanidine and carboxylic acids under elevated temperature and reduced pressure and reform upon cooling, which has been used to purify the substances in a distillation-like process. This approach is rather energy intensive but might be exploitable for recycling of ILs after cellulose processing if all volatile compounds, e.g., the non-solvent used for precipitation, degradation- and reaction side products, and other impurities are removed prior to “IL distillation”.

Imidazolium chlorides proofed to be efficient solvents for cellulose but their high melting points, viscosities, and corrosiveness limit application for cellulose processing. Thus, ILs with novel anions of high hydrogen bond basicity have been studied intensively that circumvent these drawbacks and still facilitate dissolution of cellulose. In addition to room temperature liquid imidazolium acetates, formates have been prepared that showed even lower viscosity than the corresponding acetates and chlorides [198]. Up to now, these ILs have not been studied for chemical derivatization although a direct comparison to acetate-based ILs might be very useful. Ammonium formates have been reported to dissolve cellulose and other polysaccharides and could be used for carboxymethylation [206]. Interestingly, the corresponding mono-, di-, and trichloroacetate salts could not dissolve cellulose, which might be due to their higher acidity, *i.e.*, their less pronounced ability to act as hydrogen bond acceptor. Cellulose dissolving ILs with high hydrogen bond basicities and comparably low viscosities and melting points have been obtained by combination of various imidazolium- and ammonium cations with dialkylphosphates or alkylphosphonates [113,117,123]. Substitution of one oxygen by sulfur or selenium in these anions is assumed to result in a further decrease of melting point and viscosity due to the reduced symmetry [209].

An interesting development in the field of IL research is the design of switchable solvents composed of a base (amidines, guanidines, amines) and a CO_2_-capturing group (amines, alcohols, water) [210,211]. Exposure of these systems to CO_2_ atmosphere triggers the conversion from non-ionic/hydrophobic to ionic/hydrophilic. The equilibrium can be shifted back to the original state by removal of CO_2_, e.g., via temperature increase or flushing with inert gas. This approach can become useful for IL recycling by induced phase separation from an aqueous solution. Switchable ILs prepared *in situ* from DBU, an alcohol, and CO_2_ have been tested for the extraction of hemicelluloses from spruce [212]. However, major improvements of this system are required because even after 5 days the IL could only remove about 40% the hemicelluloses and almost no cellulose from the wood material. More importantly, the process had to be performed at elevated temperature at which CO_2_ was eliminated from system. Thus, constant gas supply over the whole time was required.

As a concluding remark it has to be noted that a particular cellulose dissolving IL is not necessarily suitable for any kind of chemical derivatization. Moreover, the influence of the cation must not be neglected. Even though its influence on the dissolution mechanism is debatable, the cation may still influence physical properties and chemical behavior. As an example; room temperature liquid imidazolium dialkylphosphates could not be used for homogeneous acetylation or tosylation of cellulose because gelation of the reaction mixture occurred shortly after the addition of the derivatization reagent [20,74]. On the contrary, various cellulose esters and mixed esters were accessible in a completely homogeneous reaction using tetralkylammonium salts carrying the same anion as reaction media [20].

### 4.4. Combination of Derivatization and Shaping of Cellulose in Ionic Liquids

ILs have demonstrated to be efficient and versatile reaction media for the homogeneous derivatization of cellulose. Likewise, they have huge potential as solvents for shaping of the polysaccharide into various objects and are studied intensively for commercial applications in large scales. Combination of these two aspects, *i.e.*, derivatization of cellulose in ILs and immediate shaping of the derivatives obtained, appears to be a logical step since time and energy consuming product isolation and purification steps can be omitted or at least reduced to a minimum. Other cellulose solvents are only of limited suitability for this approach because they are either not suitable for derivatization, e.g., NMMO, CS_2_, or show poor recyclability and are therefore not efficient for the shaping process, e.g., DMA/LiCl. Only few reports have been published that describe the combination of derivatization and shaping of cellulose in ILs but it can be expected that with increasing knowledge in the field, this approach might find more interest. Without isolation and purification of the derivative, fibers of cellulose acetates with different DS values could be obtained by using the corresponding reaction mixture for the spinning processes [213]. Enzyme containing capsules with a polyelectrolyte complex shell and a porous polysaccharide core have been prepared by preparation of anionic cellulose sulfate in IL/co-solvent and subsequent *in situ* complex formation with a polycation [87].

## 5. Conclusions

Since their first description as cellulose solvents 10 years ago, ILs rapidly demonstrated their huge potential for shaping of the polysaccharide into different cellulosic materials, for fractionation of biomass and conversion into biofuels and platform chemicals, as well as for the synthesis of various cellulose derivatives with well-defined properties. Based on a critical analysis, research in this field appears to be at a tipping point. Several drawbacks of ILs became apparent that have to (and in the authors opinion will) be solved in order to exploit these solvents in cellulose-based processes. This feat will require interdisciplinary communication between experts with backgrounds in general organic chemistry, polysaccharide research, physical and theoretical chemistry, chemical- and process engineering, biochemistry, toxicology, material testing, and other related areas. Aim of the present review was to provide a basis for this discussion by giving a comprehensive overview on recent, current, and future research regarding the use of ILs as cellulose solvents in general and as reaction media for the synthesis of cellulose derivatives in particular. Thereby, the authors explicitly encourage researchers from seemingly unrelated fields to contribute their unique perspective to the ongoing discussions. As a final remark, it is proposed that efficient solvent recycling and the use of task-specific ILs will be the key issues in future developments. The later includes the design of a “next generation” of cellulose dissolving ILs as well as the more conscious use of established ones, based on their specific advantages and disadvantages.
